# Lipopolysaccharide of Yersinia pestis, the Cause of Plague: Structure, Genetics, Biological Properties

**Published:** 2012

**Authors:** Y.A. Knirel, A.P. Anisimov

**Affiliations:** Zelinsky Institute of Organic Chemistry, Russian Academy of Sciences, Leninsky prospect, 47, Moscow, Russia, 119991; State Research Center for Applied Microbiology and Biotechnology, Obolensk, Moscow Region, Russia, 142279

**Keywords:** lipopolysaccharide, lipid A, plague, *Yersinia pestis*, immune response, antibiotic resistance

## Abstract

The present review summarizes data pertaining to the composition and structure of the carbohydrate moiety (core oligosaccharide) and lipid component (lipid A) of the various forms of lipopolysaccharide (LPS), one of the major pathogenicity factors of*Yersinia pestis*, the cause of plague. The review addresses the functions and the biological significance of genes for the biosynthesis of LPS, as well as the biological properties of LPS in strains from various intraspecies groups of*Y. pestis *and their mutants, including the contribution of LPS to the resistance of bacteria to factors of the innate immunity of both insect-vectors and mammal-hosts. Special attention is paid to temperature-dependent variations in the LPS structure, their genetic control and roles in the pathogenesis of plague. The evolutionary aspect is considered based on a comparison of the structure and genetics of the LPS of*Y. pestis *and other enteric bacteria, including other*Yersinia *species. The prospects of development of live plague vaccines created on the basis of*Y. pestis *strains with the genetically modified LPS are discussed.

## INTRODUCTION

In the past decade, significant progress has been achieved in the study of the chemical structure, biosynthesis, and biological role of the lipopolysaccharide (LPS) as one of the pathogenicity factors of the bacteria *Yersinia pestis, * the cause of plague. Significant progress in this area has been achieved in the past decade after the September 2001 terrorist attacks, horrific events that prompted thorough research in dangerous pathogens that could potentially find application in the realm of biological terrorism.

The *Yersinia * genus belongs to the Enterobacteriaceae family. Unlike other representatives of this family, including two enteropathogenic * Yersinia* species, * Y. pseudotuberculosis * and *Y. enterocolitica* , which cause chronic intestinal infections, *Y. pestis * cannot exist under ambient conditions for a long time. The plague microbe circulates in natural foci, including populations of over 200 species of rodent hosts (ground squirrels, marmots, gerbils, voles, pikas, *etc.* ) and insect vectors (over 80 flea species) [1–5]. A high mortality rate due to plague in rodents is a prerequisite for the continuous transmission of *Y. pestis * in nature.

The *Y. pestis* species contains both genotypically and phenotypically different variants [5, 6]. Strains of the main subspecies, *Y. pestis * subsp. *pestis* , belonging to biovars antiqua, medievalis, orientalis, and intermedium are virulent for humans and guinea pigs. Three plague pandemics are believed to have been caused by strains of each of the first three biovars. It has been proposed that strains of biovars altaica, caucasica, hissarica, ulegeica, talassica, xilingolensis, ginghaiensis, and angola, which are highly virulent in rodent hosts (various vole species belonging to the genus * Microtus* ) and white mice, while avirulent in guinea pigs and humans, should be attributed to the second subspecies of *Y. pestis* subsp. *microtus * [6, 7]. This terminology already exists in popular usage [8], and in this review, we will adhere to this variant of intraspecies classification of the causative agent of plague.

Plague is transmitted to humans predominantly via bites of infected fleas, as well as via direct contact with damaged skin and mucous membranes or via the inhalation of aerosolized respiratory secretions from animals or humans with the pneumonic form of the infection [2–5]. In humans, plague occurs as an acute infectious disease manifesting itself as an extremely severe intoxication, fever, lesions in lymph nodes, lungs, and other internal organs, which is frequently complicated with sepsis [4].

The high pathogenicity of *Y. pestis * is to a significant extent due to the unique ability of the bacteria to overcome the defence mechanisms of both mammals and insects, thus ensuring their survival during the entire transmission cycle. Lipopolysaccharide (LPS, endotoxin), the major component of the outer membrane of the cell wall which forms the outer layer of the LPS-phospholipid bilayer, contributes significantly to this feature of the plague microbe. The lipid component of the LPS (the so-called lipid A) acts as an anchor that keeps the LPS molecule bound to the membrane, whereas its carbohydrate chain is oriented towards the environment. A number of pathogenic rough-colony-forming bacteria, including *Y. pestis* , produce R-type LPS with its carbohydrate moiety being limited to an oligosaccharide (pentasaccharide or higher), which is named the core. The S-type LPS, which is typical of most smooth-colony-forming bacteria, in addition contains a polysaccharide chain (O-antigen) consisting of oligosaccharide repeating units, whereas the core is an intermediate region between the O-antigen and lipid A.

The biosynthesis of the O-antigen and the core-lipid A region proceeds via the independent, but convergent pathways [9]. The initial stages, i.e. the synthesis of lipid A, the transfer of core components to it, and the assembly of the O-antigen repeating unit on an undecaprenol carrier, are performed on the cytoplasmic side of the inner membrane. These stages are followed by the transmembrane transfer. The subsequent stages (polymerization of the repeating unit via the O-antigen-polymerase-dependent pathway, which is the most common pathway in enterobacteria; the possible further modifications in the core-lipid A region and O-antigen; and the linking of both parts into a single molecule) occur on the periplasmic side of the membrane.

LPS plays a significant role in the resistance of bacteria to antibiotics, complement, and other defence systems of the host organism; thus, it can be considered as one of the pathogenicity factors of Gram-negative bacteria. The fine structure of the carbohydrate moiety of LPS determines the specificity of the interaction between a bacterial cell and other biological systems, including the immune system and bacteriophages. Lipid A is responsible for the majority of the physiological effects caused by LPS in animals and humans. In mammals, the molecular mechanisms of these effects include the activation of specialized host cells, such as monocytes and macrophages, via the toll-like receptor TLR4, with the participation of the LPS-binding protein and co-receptors CD14 and MD-2. The activated cells secrete nitrogen monoxide, vasoactive lipids and bioactive mediators– pro-inflammatory cytokines. Low concentrations of cytokines are required for triggering the innate immune system of the host; however, their excessive concentration leads to the septic (endotoxic) shock.

Reviews devoted to structural features of the LPS of the plague microbe [10], as well as the immunological properties of its antigens, including LPS [11], have been recently published. This review presents the most up-to-date data pertaining to the chemical composition, structure, genetics and biosynthesis of the LPS of *Y. pestis* , as well as the biological function of the LPS, considered in the context of its structural features. The review also contains a discussion on the possible ways by which the accumulated data pertaining to LPS could be applied in health care. 

## CHEMICAL COMPOSITION AND STRUCTURE

**Fig. 1:**
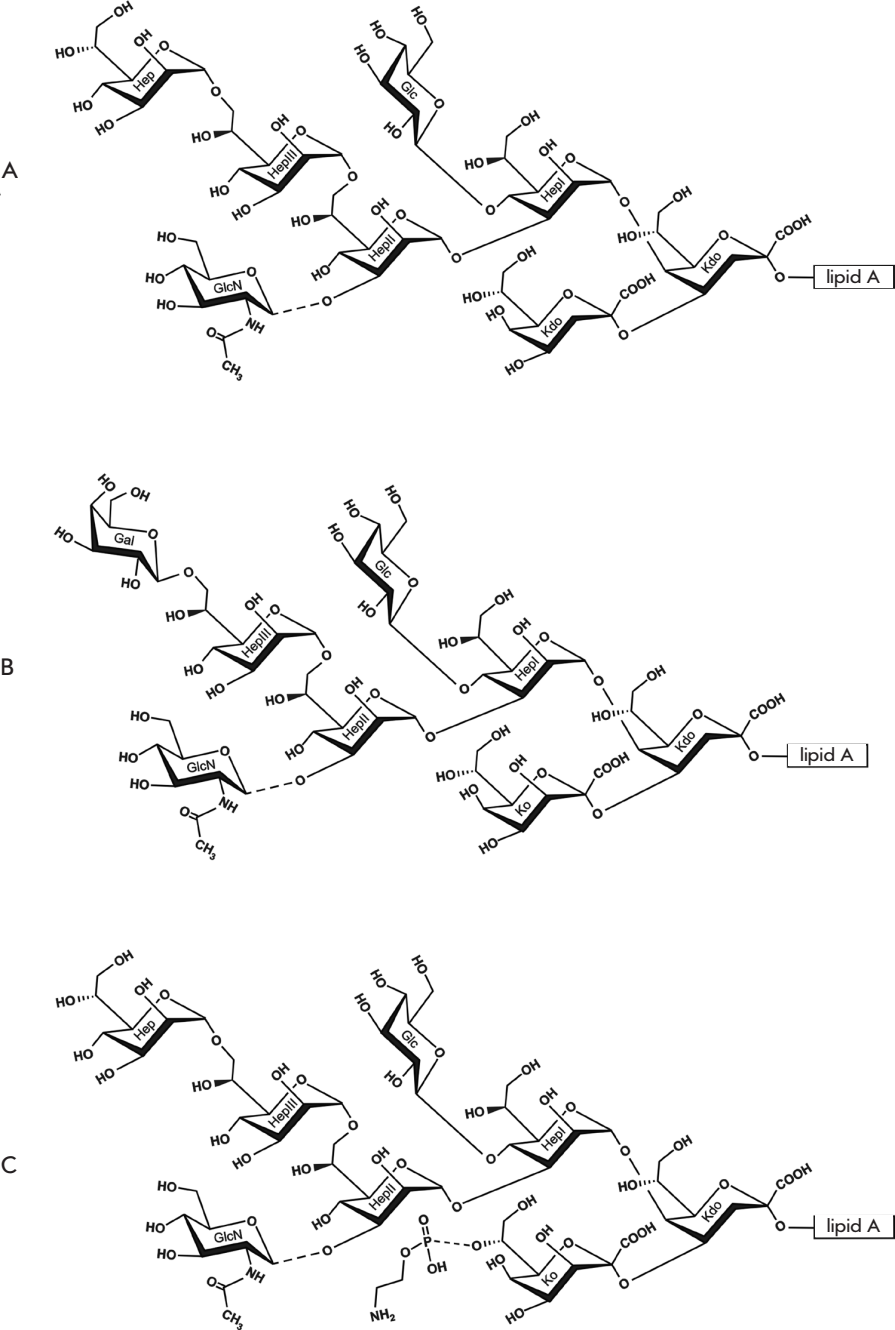
Structural variants of the LPS core of *Y. pestis* [11–13, 17]. (A) DD‑Hep+Kdo glycoform synthesized as the major variant at 37°C and one of the variants produced at 20–28°C. (B) Gal+Ko glycoform synthesized as one of the variants at 20–28°C; in addition, DD-Hep+Kdo, DD-Hep+Ko and Gal+Kdo glycoforms are present. (C) DD‑Hep+KoPEtN glycoform synthesized at 6°C; in addition, Gal+Ko PEtN-lacking glycoform is present. Dotted lines indicate nonstoichiometric substitution. Glycine located on LD-HepI in some strains is not shown.

The LPS of *Y. pestis * is composed of a short carbohydrate (oligosaccharide) chain bound to lipid A [10, 12, 13]. This chain contains a conserved pentasaccharide moiety, the so-called inner core, which is typical of all wild strains of enterobacteria. It consists of three residues of *L* - *glycero* - *D* - *manno* -heptose (LD-Hep) and two residues of 3-deoxy- *D* - *manno* -oct-2-ulosonic acid (ketodeoxyoctonic acid, Kdo) ( *Fig. 1* ). The inner core of *Y. pestis,* and certain other enterobacteria ( *Serratia, Klebsiella, Proteus, Providencia* ) [14], also contains a *D* -glucose residue bound to the heptose residue proximal to lipid A (LD-HepI). The aforementioned bacteria form the group with the so-called non-salmonella core type, whereas the salmonella core type contains phosphate, diphosphate, or diphosphoethanolamine, instead of a glucose residue, at the same position of LD-HepI [14]. The inner core region functions as a receptor of most of the bacteriophages specific to the LPS of *Y. pestis* , including bacteriophage φA1122 belonging to the T7 group [15, 16], which is used by the U.S. Center for Disease Control and Prevention for phage diagnostics of *Y. pestis* . The glycosidic bond of the Kdo residue located at the reducing terminus of the inner core binds the core to lipid A.

The structure of the *Y. pestis * LPS varies depending upon certain environmental factors; it has been thoroughly studied on preparations isolated from bacteria cultured at different temperatures, serving to imitate the conditions in warm-blooded mammals (37°С), poikilothermic insects (20–28°С), and hibernating animals (6°С). The complete inner core is synthesized by both *Y. pestis* subspecies cultured both at 20–28°С and 37°С. However, the described structure is the sole (or almost sole) glycoform only at 37°С. ( *Fig* .  *1А* ), whereas a decrease in temperature results in the partial replacement of the Kdo residue in the side chain by its isosteric 3-hydroxy derivative, the residue of *D-glycero* - *D* - *malo* -oct-2-ulosonic acid (Ko) [12] ( *Fig. 1B,C* ). The Ko-containing glycoform is predominant at 6°С [17].

*Y. pestis *lacks the outer oligosaccharide region that would be present in the salmonella core type. Its inner region is decorated with several monosaccharides and noncarbohydrate components that are typical of the *Yersinia* species. Thus, the heptose residue that is most distal from lipid A (LD-HepIII) carries a residue of *D* - *glycero* - *D* - *manno* -heptose (DD-Hep) or *D* -galactose. The former is typical of the high-temperature LPS variants ( *Fig. 1A* ), whereas both variants are synthesized at ambient and decreased temperatures [12] ( *Figs. 1A,B* ). Strains of certain biovars (caucasica *, * altaica) of *Y. pestis* subsp. *microtus * are incapable of incorporating DD-Hep into the LPS; as a result, most residues of LD-HepIII in the high-temperature LPS forms do not carry any monosaccharide substituents [12, 18, 19].

**Fig. 2:**
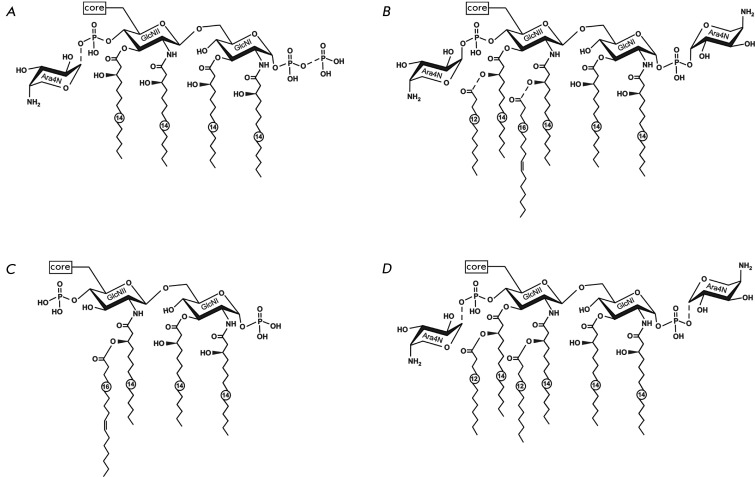
Structural variants of lipid A of *Y. pestis* . (A) Tetraacyl form synthesized by wild-type strains at 37°C [11, 12, 23, 25]. One of the variants is shown; in the other variants, a diphosphate group may occur at position 4’ and Ara4N-1-phosphate at position 1; Ara4N-1-diphosphate may replace each of these groups [25]. (B) Hexaacyl form [11, 12, 20, 23] and (C) tetraacyl form [17] synthesized by wild-type strains at 20–28°C and 6°C, respectively. (D) Hexaacyl form synthesized by a recombinant strain of *Y. pestis* bearing the *lpxL* gene of *Escherichia coli* at37°C and 26°C [27]. Dotted lines indicate nonstoichiometric substitution.

The central heptose residue (LD-HepII) is substituted with a residue of *N* -acetyl- *D-* glucosamine, which is present in nonstoichiometric amounts. One of the heptose residues (according to the authors’ unpublished data, it is LD-HepI) can be partially acylated with glycine, the content of which decreases with increasing cultivation temperature [12]. The Ко residues are nonstoichiometrically phosphorylated with phosphoethanolamine (PEtN) at 6°С [17] ( *Fig. 1C* ). PEtN is also present in certain strains grown at 25°С [18, 19].

*Y. pestis* lipid A has a carbohydrate backbone that is typical of enterobacteria and consists of two 1,4’-biphosphorylated glucosamine residues acylated with four residues of 3-hydroxymyristate, which are known as primary acyl groups. Two residues bind to the amino groups, and the two others, to the hydroxyl groups of glucosamine residues ( *Fig. 2A* ). Secondary acyl residues, laurate and palmitoleate, bind to the hydroxyl groups of primary fatty acids in the glucosamine residue carrying the core oligosaccharide (GlcNII) [12, 20] ( *Fig. 2B* ). An additional acyl residue, decanoate, has been detected in *Y. pestis* lipid A; however, its position remains unknown [21, 22].

The content of different acylated forms of lipid A depends to a significant extent on cultivation conditions: it is a mixture of tetraacyl, pentaacyl, and hexaacyl forms at 20–28°С; the triacyl form is also fairly common. A rise in temperature results in a decrease in the degree of acylation of lipid A. Thus, palmitoleate is not bound at 37°С; therefore, the hexaacyl form is not synthesized at all, and the pentaacyl form with laurate occurs only in a small amount [12, 18, 21, 23].

**Fig. 3:**
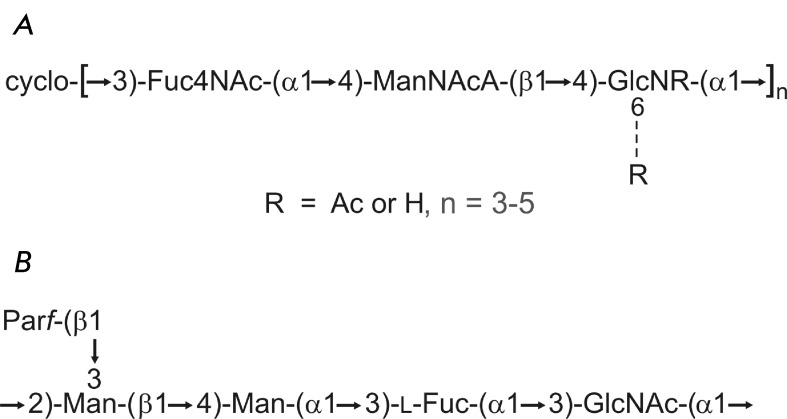
Structure of the polysaccharide antigens of *Y. pestis * (A) and *Y. pseudotuberculosis * O:1b (B). (A) The cyclic form of the common enterobacterial antigen of *Y. pestis* [26]. The glucosamine residue is N‑acetylated by ∼50 % and 6-O-acetylated by ∼20 %; n = 4 (major variant), 3 or 5 (minor variants). (B) The pentasaccharide repeating unit of the O-antigen of *Y. pseudotuberculosis* O:1b [28]. A nonfunctional gene cluster for biosynthesis of this polysaccharide is present in the genome of *Y. pestis* [29]. Par represents 3,6-dideoxy *-D* - *ribo* -hexose (paratose). All monosaccharides have the *D* configuration; paratose occurs in the furanose form, and the other monosaccharides occur in the pyranose form.

The high-temperature tetraacyl form (the so-called lipid IV _A_ ) contains four primary residues of 3-hydroxymyristate ( *Fig. 2A* ), whereas another tetraacyl form with three residues of 3-hydroxymyristate, one of which carries palmitoleate [17] ( *Fig. 2B* ), is produced at a decreased temperature (6°C), along with the hexaacyl form ( *Fig. 2B* ). Another feature of *Y. pestis* lipid A is the cold shock-induced oxidation of one or two of the acyl groups [17]. However, it remains unclear which fatty acids are oxidized and which hydroxylated derivatives are formed during this process.

The phosphate groups of lipid A are glycosylated with residues of a cationic monosaccharide, 4-amino-4-deoxy- *L* -arabinose (Ara4N). In low-temperature LPS variants, the glycosylation of both phosphate groups is almost stoichiometric ( *Fig. 2B* ), whereas the Ara4N content decreases in high-temperature forms [12, 21], and one of the phosphate groups is additionally phosphorylated giving rise to a diphosphate group [18, 19, 24, 25] ( *Fig. 2A* ). In tetraacyl lipid A of bacteria cultured at 37°С, the diphosphate group can be located at any of two possible positions. Its presence in the pentaacyl variant at position 4’ has been confirmed [25], but its location at position 1 also cannot be excluded. By approximate estimation based on mass spectrometry data, the total diphosphate content in the tetraacyl form is 5–6%. Similarly to monophosphate groups, the diphosphate groups at both positions of lipid A can be partially glycosylated with Ara4N [25]. LPS with the PEtN residue in the core, which is produced at 6°C, contains no Ara4N in lipid A [17].

The lack of any O-antigen polysaccharide chain is a feature of the *Y. pestis* LPS that distinguishes it from the LPS of other yersiniae. Meanwhile, similarly to other enterobacteria, *Y. pestis * can produce the enterobacterial common antigen polysaccharide composed of trisaccharide repeating units comprising one residue of each of the following compounds: *N* -acetyl- *D* -glucosamine (GlcNAc), 2-acetamido-2-deoxy- *D* -mannuronic acid (ManNAcA), and 4-acetamido-4-deoxy- *D* -fucose (Fuc4NAc), the GlcNAc residue being partially O-acetylated and partially N-deacetylated. From the two known forms of this polysaccharide (the linear form bound to phospholipid or lipid A and the lipid-free cyclic form), in *Y. pestis * the most thoroughly characterized is the cyclic form [26] ( *Fig. 3А* ).

## GENETICS AND BIOSYNTHESIS

The tetraacyl biphosphorylated precursor of enterobacterial lipid A (lipid IV _A_ ) in *Y. pestis * is presumed to be synthesized via the same pathway as that of *E. coli* and *Salmonella enterica * [9], which have been studied most thoroughly in this context. Homologues of the *E. coli* genes that encode the enzymes mediating the late acylation of lipid A (myristoyltransferase LpxM (MsbB), palmitoleyltransferase LpxP and palmitoyltransferase PagP but not lauroyltransferase LpxL (HtrB)), have been identified in the *Y. pestis * genome [22, 30–32].

**Fig. 4:**
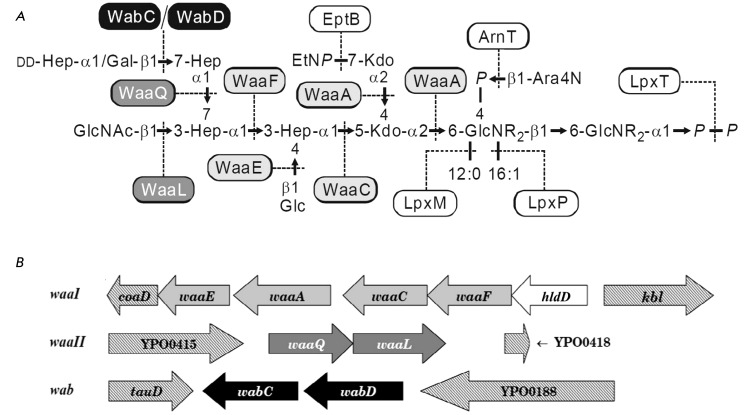
Biosynthesis of the LPS of *Y. pestis* [22, 32–34]. (A) Enzymes of the LPS core synthesis and the late stages of the lipid A synthesis. R represents 3‑hydroxymyristoyl. (B) Organization of the gene clusters for the LPS core synthesis. The genes in the clusters *waaI * (YP0054-YP0058), *waaII* (YP0416-YP0417), and *wab * (YP0186-YP0187) and the corresponding glycosyl transferases are shown in light gray, dark gray, and black, respectively. The functions of the glycosyl transferases genes are assigned based on the analysis of available *Y. pestis* full genome sequences and the data on LPS structure in knockout mutants at each gene. The figure is reproduced from the paper of the authors [32] with the permission of the Nauka Publishing Company.

The functional *lpxM* and *lpxP* genes participate in the synthesis of the hexaacyl lipid A of *Y. pestis * ( *Fig. 4* ). Their expression level increases as the cultivation temperature decreases from 37 to 21°С. Regardless of temperature, the mutant at both genes synthesizes tetraacyl lipid A, which is similar to lipid IV _A_ ( *Fig. 2A* ) and is identical to that synthesized by wild-type *Y. pestis * strains at 37°С [22]. Meanwhile, the transcription level remains low under all conditions. The temperature dependence of the catalytic activity of the enzymes or other post-transcription effects can also affect the lipid A acylation pattern.

*E. coli* acyltransferase LpxM can use either myristate or laurate as a substrate; however, since its activity is higher with the former substrate, it links myristate to 3-hydroxymyristate at position 3’ of GlcNII. This process is preceded by the transfer to 3-hydroxymyristate at position 2’ of GlcNII of the secondary acyl group: laurate at 30–42°С catalyzed by LpxL [9] or palmitoleate at cold shock temperature (12°С) with the participation of LpxP [35]. The *Y. pestis* homologues LpxM and LpxPtransfer laurate and palmitoleate to 3-hydroxymyristate residues at positions 3’ and 2’ GlcNII, respectively. Temperature control was observed only for LpxP, transferring palmitoleate to position 2’ GlcNII prior to the binding of the secondary acyl group at position 3’ (laurate in *Y. pestis* or myristate in  *E. coli* ), whereas LpxМ activity is temperature-independent. Since *Y. pestis * lacks the *lpxL* gene, 3-hydroxymyristate at position 2’ remains unsubstituted at an increased temperature, which deteriorates the efficiency of the laurate transfer with LpxM and results in the synthesis of mostly the tetraacyl form of lipid A and only a negligible amount of the pentaacyl form. The evidence for this is that the *Y. pestis * recombinant strain KIM5-pLpxL carrying the *E. coli lpxL* gene produces the hexaacyl form of lipid A with two secondary laurate residues both at 37 and 26°С [27] ( *Fig. 2D* ).

Acyltransferase PagP in *E. coli * and *S.* *enterica * transfers palmitate from position sn-1 of the glycerophospholipid [36], which distinguishes it from the early and other late acyltransferases that use a substrate bound to the acyl-carrier protein as a donor. Furthermore, palmitoylation of lipid A occurs on the outer rather than the inner membrane [36]. The palmitoylated forms of lipid A are also typical of *Y. pseudotuberculosis* and *Y. enterocolitica* [19–21] but have not been found in wild-type *Y. pestis* strains, despite the presence of the *pagP* homologue in the genome, which is 99% identical to the *Y. pseudotuberculosis * gene [31, 36]. The reason for this is the inactivation of this gene due to the substitution of a single nucleotide, resulting in the conversion of a tryptophan-200 codon into a stop codon [31].

The genes encoding the synthesis and transfer of Ara4N are components of the operon *arn * ( *pmrHFIJKLM* ) [9, 37]. An undecaprenyl phosphate (UndP) derivative synthesized with the participation of Ara4N-transferase ArnC (PmrF) is a donor of Ara4N for its subsequent binding to the phosphate groups of lipid A. The transfer of Ara4N to lipid A, catalyzed by the product of the *arnT* ( *pmrК* ) gene, occurs on the periplasmic side of the inner membrane [38]. A complete inner core is required to ensure the most efficient binding of Ara4N, whereas the presence or absence of core monosaccharides distal from lipid A (GlcNAc, Gal and DD-Hep) has virtually no effect on this process [32–34]. Similar to that in *E. coli* and *S. enterica,* the *arn * operon in *Y. pestis* is regulated by two-component signal transduction systems, PhoP/PhoQ and PmrA/PmrB [21, 39]. However, in *Y. pestis * the mechanism of regulation by the PhoP/PhoQ system differs as it occurs without the participation of the PmrD protein, which is absent in this bacteria [39].

The synthesis of the *E. coli * core oligosaccharide begins with the attachment of two Kdo residues to lipid IV _A_ , a process that is catalyzed by bifunctional Kdo transferase WaaA ( *Fig. 4А* ). Kdo transfer precedes the late acylation of lipid A [9]. The subsequent assembly of the core takes place on a completely acylated lipid A and is followed by the transfer of LPS consisting of the core and lipid A through the inner membrane, with the aid of the ABC transporter MsbA. Meanwhile, neither core oligosaccharide nor Kdo (Ko) residues are required for the transmembrane transfer, since LPS without any core (i.e., lipid A) is expressed in the Kdo-deficient mutants of *E. coli* [40, 41] and *Y. pestis * [32, 33, 42].

The core biosynthesis genes in *E. coli* , *S. enterica* , and a number of other enterobacteria are clustered in a chromosomal region, forming the *waa* cluster [9]. Two clusters ( *waaI * and *waaII* ) with four and two homologues of the *waa * genes and one cluster with two *wab* genes, which also encode core biosynthetic enzymes, have been identified in the *Y. pestis* genome [32–34] ( *Fig. 4B* ).

The *waaI * cluster containing most of the genes for the synthesis of the inner core comprises the genes of Kdo transferase WaaA; heptosyltransferases WaaC and WaaF to transfer LD-HepI and LD-HepII, respectively; and glucosyltransferase WaaE. In addition, it includes the gene of heptose 6-epimerase HldD catalyzing the synthesis of ADP-LD-Hep from its biosynthetic precursor ADP-DD-Hep. Yet another heptosyltransferase gene, *waaQ* , is located in the *waaII* cluster. The enzyme encoded by this gene transfers LD-HepIII to LD-HepII; glucose must be present on LD-HepI in order to accomplish the transfer. In turn, the glucose transfer requires the prior attachment of LD-HepII.

The second gene in the cluster *waaII* is a homologue of the gene of ligase WaaL, which attaches the O-antigen to the core [9]. Unlike monosaccharide LPS components transferred by glycosyltransferases in the form of proper nucleoside diphosphate derivatives or (in case of Kdo) a nucleoside monophosphate derivative, the undecaprenyl diphosphate (UndPP) derivative acts as a ligase substrate. In the absence of any O-antigen in *Y. pestis* , WaaL attaches the GlcNAc residue to the core; therefore, this residue is not a true component of the core [12]. Ligase nonstrictly depends on the attachment of Glc and LD-HepIII; without them, the efficiency of the GlcNAc transfer decreases and the LPS of the *waaE* and *waaQ* knockout mutants contains only a small amount of this monosaccharide [32, 33]. In *Y. pestis * and other enterobacteria, the *wecA* gene participating in the synthesis of the UndPP-bound GlcNAc is located in the gene cluster of the enterobacterial common antigen [43], whose biosynthesis (in the same manner to that of GlcNAc-containing O-antigens) is initiated by the transfer of GlcNAc-1-phosphate from UDP-GlcNAc to UndP.

The * wab* cluster contains the genes of glycosyltransferases WabC and WabD for the transfer of DD-Hep and Gal, respectively. It has been demonstrated using BLAST search that in strains of *Y. pestis* subsp. *microtus* bv. caucasica Pestoides F and bv. xilingolensis 91001, the * wabC* gene contains mutations resulting in disturbance of the synthesis of the corresponding protein [32]. Similar mutations are presumably present in the other DD-Hep-defective strains of *Y. pestis * subsp. *microtus* bv. caucasica and bv. altaica. The expression of the *wabD* gene and/or the activity of the WabD enzyme are temperature-dependent, and the transfer of Gal proceeds inefficiently at increased temperatures. The inability of the *phoP* mutant to incorporate Gal into the core demonstrates that galactosylation is controlled by the PhoP/PhoQ two-component signal transduction system [44]. Meanwhile, the attachment of DD-Hep does not require the functional PhoP/PhoQ system.

As mentioned above, the terminal Kdo residue at low temperature is partially substituted for the Ko residue. The latter is synthesized via the oxidation of the 3-deoxy group of Kdo with a unique Fe ^2+^ /α-ketoglutarate/O _2_ -dependent Kdo-3-hydroxylase (KdoO) [45]. Its substrate specificity has not been studied; however, taking into account the fact that KdoO is a peripheral membrane protein, it can reasonably be assumed that 3-hydroxylation of Kdo occurs on the cytoplasmic side of the inner membrane after two Kdo residues have bound to lipid A. The molecular mechanism of the modulation of the temperature-dependent Ko content in the core has as yet to be elucidated.

Gene homologues of transferase EptB (YhjW) transferring PEtN from phosphatidylethanolamine to Kdo [32, 33, 46] and phosphatase LpxT (YeiU) transferring phosphate from UndPP to lipid A giving rise to diphosphate [32, 33, 47] have also been found in the genome of *Y. pestis* [32, 33]. These genes, in an identical manner to the *kdoO* gene encoding Kdo-3-hydroxylase, as well as the genes of the late stages of lipid A synthesis (acylation and glycosylation with Ara4N), are spread over the chromosome as individual nonclustered genes. No gene of acyltransferase, which participates in glycine transfer to the LD-HepI residue, has thus far been identified in the genome of *Y. pestis* .

There is 100% homology of the proteins participating in the LPS biosynthesis (with the exception of the *wabC* gene that is mutated in a number of representatives of the nonmain subspecies; see above) within the *Y. pestis* species and 98–100% homology inside the *Yersinia * genus [32, 33]. This correlates with the high degree of similarity between the core and lipid A structures of LPS in various *Yersinia* species [19, 20]. In distantly related bacteria, the homology of the proteins WaaA, WaaC, WaaE, WaaF,EptB, LpxM, LpxP, and ArnT is beyond 70%. Meanwhile, in the enzymes WaaQ, WabC, WaaL, and KdoO, it is lower than 64%, whereas the homology between galactosyltransferase WabD that is unique to the *Yersinia* species and glycosyltransferases of other bacteria is less than 43%. The high homology of most of the proteins implicated in the LPS biosynthesis in *Y. pestis* and the bacteria belonging to various phylogenetic groups, in combination with the dispersed location of the corresponding genes in the chromosome of the plague microbe, suggests a multi-stage horizontal transfer of these genes to the genome of the *Yersinia* progenitor.

A nonfunctional O-antigen gene cluster was identified in the *Y. pestis* genome [29, 48]. At the nucleotide sequence level, it was 98.9% identical to the O-antigen cluster in *Y. pseudotuberculosis * O:1b [29] (the O-antigen structure is shown in *Fig. 3B* ). Therefore, *Y. pseudotuberculosis * O:1b is considered as the most plausible progenitor clone of *Y. pestis* . Of the 17 biosynthetic genes that have been identified in the O-antigen gene cluster of *Y. pseudotuberculosis* O:1b, five genes in the *Y. pestis * cluster are inactivated by insertions or deletions. These genes include those responsible for the synthesis of the nucleotide-activated derivatives of *L* -fucose and 3,6-dideoxy- *D* - *ribo* -hexose (paratose), precursors of the O-antigen components, in the absence of which the O-antigen synthesis is rendered impossible. It is noteworthy that while 16 genes in the clusters of two bacteria are 99–100% identical, the *wzx * gene is only 90.4% identical. This gene encodes flippase Wzx, which mediates the transmembrane transfer of the UndPP-bound pentasaccharide repeating unit of the O-antigen in *Y. pseudotuberculosis* O:1b. After the loss of this function, *Y. pestis * flippase presumably changed and centered on the transfer of a single UndPP-bound GlcNAc residue through the inner membrane, which subsequently is bound to the LPS core by ligase WaaL at the same position as the polysaccharide O-antigen in *Y. pseudotuberculosis* .

## BIOLOGICAL PROPERTIES AND ROLE IN PLAGUE PATHOGENESIS

Production by macrophages and other immune cells of the key pro-inflammatory cytokines (including the tumor necrosis factor α (TNF-α), the major mediator of septic shock (endotoxemia) that is induced by the action of LPS) plays a significant role in overcoming infectious diseases. In *Y. pestis* , as in the other Gram-negative bacteria, the cytokine-inducing activity of the LPS mediated by the TLR4 receptor is determined by the lipid A structure [49]. Thus, the production of TNF-α by human and mouse macrophage cell lines considerably falls with a decreasing degree of acylation of lipid A, in particular, with the absence of the hexaacyl form and a significant decrease in the content of the pentaacyl form [18, 23]. These structural changes in lipid A are observed when the temperature of bacterial cultivation increases from 21–28°С to 37°С, which imitates a transition from the temperature conditions in poikilothermic fleas (< 30°С) to those in warm-blooded mammals (37°С) [21, 23, 32, 33]. In terms of the TNF-α inducing activity at 25°С, LPS of *lpxM* knockout mutants occupies an intermediate position between the LPS of the parental strains cultivated at 25 and 37°С, which is in good agreement with the degree of acylation of lipid A [50].

The limited biological activity of the high-temperature low-acyl form of *Y. pestis* LPS may play a crucial role in overcoming the defence mechanisms of warm-blooded animals by bacteria. Whereas the innate immune system is efficiently stimulated by high-acyl LPS forms, the low-acyl forms are not recognized by the TLR4 receptor and, as a result, do no activate the innate immunity via the MD-2-TLR4-dependent pathway. Moreover, in the experiments with human macrophage cell lines [51] and dendritic cells [52], LPS from *Y. pestis* cells cultivated at 37°C behaved as an antagonist actively suppressing the TLR4-dependent pro-inflammatory response. The significance of this feature of LPS as a pathogenicity factor of the plague microbe has been convincingly supported by the study of the recombinant * Y. pestis* strain KIM5-pLpxL carrying the *E. coli* *lpxL* gene [27]. The LPS of this strain with the ‘unnatural’ hexaacyl lipid A ( *Fig. 2D* ) under all temperature conditions, including 37°С, stimulates the signalling via TLR4 and the induction of cytokines (TNF-α, interleukines-6 and -8) much more efficiently as compared to LPS of wild-type strains.

Remarkably, the *Y. pestis * recombinant strain KIM5-pLpxL could not cause bubonic plague in mice, despite the fact that the other pathogenicity factors, such as the type III secretion system, resistance to the bactericidal action of normal serum, and the proteolytic activity of Pla, were not affected. These findings provide a good illustration of the fact that the active (endotoxic) LPS form also plays a positive role in a host, ensuring prompt pathogen recognition and activation of the innate immune system at the early stages of infection. It has been demonstrated on mice model that attenuated *Y. pestis * strains with the immunostimulating LPS form can be regarded as a prototype for a new, efficient live vaccine against plague [27, 53].

It should be mentioned that the production of higher acylated low-temperature LPS forms is not a prerequisite for the survival of *Y. pestis * in flea intestine. Thus, the *lpxP/lpxM* double mutant with tetraacyl lipid A could colonize the digestive tract and block the proventriculus of rat flea *Xenopsylla cheopis* with the same efficiency as the wild-type strain, which is distinguished by a high level of expression of hexaacyl lipid A in a flea’s organism [22].

A decrease in the degree of acylation of lipid A with increasing cultivation temperature [22] or inactivation of the *lpxM* gene [20] resulted in a moderate or negligible reduction in the lethal toxicity of LPS preparations on the model of mice sensibilized with actinomycin D. The incapacity of the *lpxM* mutant of *Y. pestis* wild-type strain 231 to synthesize hexaacyl lipid A had no effect on its virulence, whereas the same mutation in the attenuated vaccine strain of *Y. pestis* , EV line NIIEG, reduced its ability to cause lethal infection in mice and guinea pigs [49, 54]. Importantly, a decrease in the virulence of the *lpxM* mutant of the vaccine strain was accompanied by a significant increase in its protective activity against bubonic plague as compared with the parental vaccine strain [49, 54]. This phenomenon can presumably be attributed to the pleiotropic effects of mutation, including changes in the biosynthesis and the exposure character of the major immunoreactive antigens of the bacterial cell surface [55]. If the changes between the mouse and human LPS receptor do not level these changes, inactivation of the *lpxM* gene can be used for development of a live plague vaccine with reduced adverse effects.

An increase in the cultivation temperature of *Y. pestis * and *Y. pseudotuberculosis * from 26 to 37°С resulted in an increase in the permeability of the outer membrane for the hydrophobic agent *N* -phenyl-1-naphthylamine, which correlated with a decrease in the number (and as a result, with an increase in the flexibility) of the LPS acyl chains [56]. The absence of laurate and palmitoleate made the *lpxP/lpxM* double mutant sensitive to the deoxycholate detergent, with no effect on its resistance to the hydrophobic antibiotics rifampin and vancomycin [22]. Controversial data have been obtained using cationic antimicrobial peptides (CAMPs), one of the key factors of innate immunity: a decrease in the degree of acylation did not affect the resistance to polymyxin B, but it increased the sensitivity to cecropin A [22].

Yet, the resistance to CAMPs depends on the Ara4N content in *Y. pestis* LPS. This correlation, which is also typical of *S. enterica * and a number of other bacteria [37], can be attributed to the electrostatic repulsion of CAMPs by the cationic monosaccharide, which impedes the binding between the antibiotic molecule and the negatively charged (e.g., phosphate) groups on the outer membrane. A high resistance of the wild-type *Y. pestis * strains with a near-stoichiometric content of Ara4N in the LPS (two Ara4N residues per molecule), which is attained by culturing bacteria at 20–28°С, has been demonstrated using the polymyxin B model. A decrease in the resistance to CAMP correlates with a noticeable drop in the content of Ara4N as the temperature is increased to 37°С [21, 57]. Mutants with knockout genes *galU* encoding the pathway of Ara4N synthesis [58], *arnT* [32–34] encoding Ara4N-transferase, or *phoP* [21, 44] modulating the binding of Ara4N to lipid A are sensitive to CAMP independently of the cultivation temperature. The role of Ara4N is also supported by a marked increase in the content of this monosaccharide in the LPS of bacteria cultivated at 37°С in the presence of polymyxin B [12]. An increase in the Ara4N content in LPS and, as a result, in the resistance of *Y. pestis * to CAMPs with a decreasing cultivation temperature is undoubtedly of adaptive character. High resistance to polymyxin B at a temperature characteristic of insects can presumably be attributed to a greater contribution of CAMPs to the innate immunity defence mechanisms of insects as compared to that of mammals, which have a complement system in addition to CAMPs.

Another cationic component of LPS, glycine [57] located in the core, can contribute to a certain extent to the resistance to CAMPs, whereas uncharged core components do not seem to play a significant role. An increase in the sensitivity to polymyxin B, which was observed for a set of knockout mutants at glycosyltransferase genes producing a truncated core could presumably be attributed to the simultaneous decrease in the Ara4N content in lipid A due to the inefficiency of the Ara4N transfer to LPS molecules with an incomplete carbohydrate moiety [32–34].

The LPS core plays a significant role in the resistance of *Y. pestis * to the complement-mediated bactericidal effect of normal blood serum [32–34], another important component of the innate immune system. Wild-type strains of *Y. pestis * subsp. *pestis * are resistant to normal human serum (NHS) both at 25°С and at 37°С [57]. The *waaL* , *wabC* , *wabD, * and *arnT* mutants lacking the terminal core substituents GlcNAc, DD-Hep, Gal or the cationic monosaccharide Ara4N, respectively, are characterized by an almost identical resistance. On the contrary, the mutants with an incomplete inner core region are highly susceptible to NHS [32–34]. The molecular mechanism, by which the core contributes to the serum resistance, has not been elucidated thus far. Possibly, it is mediated by the effect of LPS on the folding correctness and, as a result, on the functional activity of the outer membrane protein Ail (OmpX) [59], which plays a crucial role in the resistance of *Y. pestis * to serum [59, 60]. Studies of the recombinant *E. coli * strain carrying the *Y. pestis ompX* gene and its three mutants with a truncated core revealed that the size of the LPS core impacts not only the resistance to NHS, but also the OmpX-mediated virulence factors, such as the adhesive ability and the invasiveness of bacteria [60].

As opposed to strains of the main subspecies, the sensitivity of strains of *Y. pestis * subsp. *microtus * bv. caucasica to the action of NHS is temperature-independent, which correlates with the absence of documented cases of a human plague caused by strains of this biovar [57]. Meanwhile, these strains are resistant to mouse serum and probably to that of their main host, the common vole, ensuring their survival in rodent blood, which is required for circulation of *Y. pestis* in the natural plague foci. The only distinguishing feature of the LPS of biovar caucasica is that the core contains no DD-Hep [12]. However, a strain of another representative of *Y. pestis * nonmain subspecies, biovar altaica [57], whose LPS also contains no DD-Hep, is as highly resistant to the bactericidal effect of NHS as that of the *wabC * mutant of the main subspecies with a DD-Hep-deficient LPS [32–34]. This finding suggests that the adaptive changes, which made strains of *Y. pestis* subsp. *microtus * bv. caucasica sensitive to NHS, affected not only LPS, but also another factor(s) involved within the interaction between a bacterial cell and the complement system.

With no effect on the growth rate of *Y. pestis * cells [32, 33], a decrease in the size of the LPS core affects the *in vivo * formation of a biofilm, the polysaccharide-containing extracellular matrix, as well as the proventriculus blockage in fleas, which depends on this process [61]. A *Y. pestis * KIM6+ mutant with the knockout *gmhA* gene, which encodes one of the enzymes of the LD-Hep biosynthetic pathway, is characterized by a reduced ability to form the biofilm on the cuticle of nematode *Caenorhabditis elegans * and to block the proventriculus in *X. cheopis* with a moderate decrease in the level of *in vitro* biofilm formation [61]. This indirect effect of the absence of the major part of the core, including the heptose region, can be attributed to the interaction between the LPS core components and the outer membrane proteins participating in the synthesis, processing, or export of the biofilm.

A significant decrease in the virulence of *Y. pestis* 231 was observed in subcutaneously infected guinea pigs after the LPS core was shortened to five monosaccharide residues and the full loss of virulence for both guinea pigs and mice after the subsequent shortening of the LPS core to three monosaccharides [32, 33]. However, these observations were carried out for a period of 21 days, and it cannot be excluded that a prolongation of the experimental time could have led to the generalization of the infection, resulting in animal death at later stages. Attenuation of the mutants of the virulent CO92 strain of *Y. pestis * with a truncated core was also observed in BALB/c mice, whereas the absence of the core in mutants at the * yrbH* gene of the Kdo synthesis pathway or the *waaA * gene encoding Kdo transferase made them completely avirulent [15]. These data prove directly the exceptional significance of the LPS for the pathogenicity of the plague microbe, since an identical dramatic attenuation of *Y. pestis * strains was observed only when the main components of the type III secretion system [4], the gene cluster of synthesis and reception of the siderophore yersiniabactin [4], or the lipoprotein NlpD-encoding genes [62] were lost.

The biological significance of temperature-dependent variations in the monosaccharide composition of the core (replacement of the terminal Kdo and DD-Hep residues for the Ko and Gal residues, respectively, with decreasing ambient temperature from 37 to 28°С and lower) remains to be elucidated. It is reasonable to assume that the hydroxylation of Kdo at a reduced temperature (i.e., the conversion of Kdo into Ko) compensates for the decrease in the hydrophilicity of the LPS as a result of the acylation of the hydroxyl groups of primary fatty acid residues, which also occurs at low temperatures.

The investigation of the plasminogen activator Pla of *Y. pestis * (an outer membrane protein belonging to the omptin family and exhibiting functions of protease/adhesin) provided an answer (which may not be the only answer) to the intriguing question of which other advantages (in addition to the trivial energy saving advantage) was acquired by the plague microbe after it had eliminated the necessity for synthesizing the O-antigen. Pla converts plasminogen into plasmin, a key fibrinolytic enzyme, destroys the circulating α _2_ -plasmin inhibitor (antiplasmin), induces an uncontrollable tissue proteolysis, and facilitates the dissemination of *Y. pestis * in a macroorganism, thus playing a significant role in a plague pathogenesis. Studies of the activity of *Y. pestis* Pla in recombinant *Y. pseudotuberculosis* strains with different levels of the O-antigen expression have demonstrated that the O-antigen sterically impedes the interaction between Pla and the high-molecular-mass substrate, thus impeding both plasminogen activation and α _2_ -antiplasmin inactivation [63, 64]. This fact leads to the conclusion that the loss of the O-antigen, which is necessary for the enhancement of the enzymatic activity of Pla, has enhanced the invasiveness of *Y. pestis* . On the other hand, the Pla activity depends on a specific interaction with the phosphate groups of lipid A [65] and requires the presence of LPS with a core that contains at least two LD-Hep residues [32, 66]. Such an unusual LPS dependence can be attributed to the fine conformational changes in the active site of omptin due to the binding with LPS [67].

## CONCLUSIONS

A comparison of data pertaining to the structure, biosynthesis, and the biological properties of the LPS in *Y. pestis * and *Y. pseudotuberculosis* demonstrates that inactivation of the O-antigen gene cluster and the *pagP * gene, as well as the loss of the *lpxL* gene, which results in the synthesis of the R-form of LPS with a short carbohydrate chain and a loss of the ability to produce high-acyl forms of lipid A at 37°С, were the most significant events during the evolution of *Y. pestis* associated with changes in the LPS structure. These changes play a significant role in the plague pathogenesis, being an important part of the strategy by which the bacteria overcome the host’s defence mechanisms. Thus, the absence of the polysaccharide chain ensures the functioning of such a significant pathogenicity factor of *Y. рestis* as the plasminogen activator. The temperature-mediated decrease in the degree of LPS acylation resulting in a reduction of the cytokine-inducing ability is considered to be one of the mechanisms for the prevention of pathogen recognition by the host’s immune system at the early stages of infection. These data agree with the conception that the pathoadaptation of *Y. pestis * to a new ecological niche included the loss of the functionality of a number of genes required for saprophytic existence [36, 68]. The mutations in the *wabC * gene encoding DD-Hep-transferase, which remained in representatives of bv. caucasica and bv.xilingolensis of the nonmain subspecies *Y. pestis * subsp. *microtus* , may have been an element of the subsequent reductive intra-species microevolution during the adaptation of *Y. pestis* to circulation in the populations of certain vole species.

Meanwhile, a number of LPS features, such as the temperature-dependent variation of both core and lipid A structures, were inherited by *Y. pestis* from *Y. pseudotuberculosis * without any noticeable changes. Some of these variations, such as the phosphorylation with PEtN, the glycosylation with Ara4N, and the oxidation of Kdo into Ko, presumably do not possess any fundamental significance in the new lifestyle of the plague microbe and are required only for the normal functioning of the outer membrane, which is achieved by imparting a certain hydrophilicity and a certain charge to it. On the other hand, they may contribute to plague pathogenesis by facilitating the optimal adaptation of *Y. pestis * at different phases of its existence under the considerably different conditions in mammal hosts and insect vectors. The unique phenomenon of the plague microbe and plague can be a result of the synergic effect of the inherited and newly acquired pathogenicity factors, including those associated with LPS.

Thus, the recent data highlight LPS as a multifunctional pathogenicity factor of *Y. pestis * which plays a key role in the adaptive variability of the plague microbe. However, it should be noted that even if there is a correlation between the LPS structure and the properties of the bacterial culture, the exact biological significance of various LPS modifications cannot be considered completely ascertained, since the cultivation of bacteria in laboratory conditions cannot simulate exactly *in vivo* conditions. If detailed structures of the LPS synthesized by the bacteria in flea and a warm-blooded host were identified, this would enable selection of the *in vitro* conditions necessary for production of the LPS forms characteristic of a particular infected animal for further laboratory experiments.

The identification of the structure-to-function relationships in * Y. pestis* LPS opens up new prospects for the design of efficacious live vaccines against plague which are based on the attenuated strains with reduced adverse effects. The approach based on genetic-engineering modification of the *Y. рestis* LPS, which reduces the degree of lipid A acylation, is highly promising. The ability to avoid recognition by the host’s defence system at the earliest stage of infection allows the attenuated strain with the mutant LPS to rapidly multiply. However, the absence of one of the major pathogenicity factors as a result of a mutation used for attenuation of a *Y. рestis* strain prevents the generalization of the infection. This subsequently ensures efficient production of antibodies against the major antigens of the plague microbe and the unset of acquired immunity. Development of efficacious and highly selective next-generation antimicrobial preparations based on high-affinity oligonucleotide ligands can become an alternative approach in plague treatment. Understanding the genetic control of the key stages of the biosynthesis of LPS, intervention in which disturbs the functioning of the outer membrane and attenuates the effect of other virulence factors, will enable to propose new molecular targets for these antibiotics. 

## Abbreviations

CAMP: cationic antimicrobial peptides
LPS: lipopolysaccharide
NHS: normal human serum
Ara4N: 4-amino-4-deoxy-*L*-arabinose
Gal: galactose
Glc: glucose
GlcN, GlcNAc: glucosamine,*N*-acetylglucosamine
DD-Hep, LD-Hep: *D*-*glycero*-,*L*-*glycero*-*D*-*manno*-heptose
Kdo: 3-deoxy-*D*-*manno*-oct-2-ulosonic acid
Ko: *D*‑*glycero*-*D*-*talo*-oct-2-ulosonic acid
PEtN: phosphoethanolamine
UndP, UndPP: undecaprenyl phosphate, diphosphate
